# Piperidinium bis­(2-oxidobenzoato-κ^2^ *O* ^1^,*O* ^2^)borate

**DOI:** 10.1107/S1600536808042608

**Published:** 2008-12-20

**Authors:** Zhi-Hua Tang, Chaojun Huang

**Affiliations:** a School of Chemistry & Environmental Science, Shaanxi University of Technology, Hanzhong, Shaanxi Province 723001, People’s Republic of China; b Department of Physics, Shaanxi University of Technology, Hanzhong, Shaanxi Province 723001, People’s Republic of China

## Abstract

The asymmetric unit of the title compound, C_5_H_12_N^+^·C_14_H_8_BO_6_^−^ or [C_5_H_12_N][BO_4_(C_7_H_4_O)_2_], contains two piperidinium cations and two bis­(salicylato)borate anions. The coordination geometries around the B atoms are distorted tetra­hedral. In the two mol­ecules, the aromatic rings are oriented at dihedral angles of 76.27 (3) and 83.86 (3)°. The rings containing B atoms have twist-boat conformations, while the two cations adopt chair conformations. In the crystal, the component species are linked by N—H⋯O hydrogen bonds. In the crystal structure, intra- and inter­molecular N—H⋯O hydrogen bonds link the mol­ecules.

## Related literature

For general background, see: Barthel *et al.* (2000 ▶); Downard *et al.* (2002 ▶). For related structures, see: Han *et al.* (2007 ▶); Li & Liu (2006 ▶); Zhang *et al.* (2005 ▶). For ring puckering parameters, see: Cremer & Pople (1975 ▶).
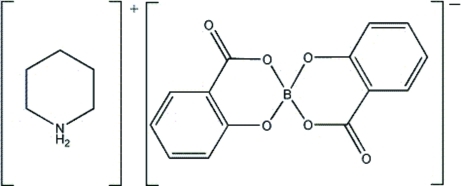


## Experimental

### 

#### Crystal data

C_5_H_12_N^+^·C_14_H_8_BO_6_^−^
*M* *_r_* = 369.17
Monoclinic, 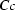

*a* = 19.835 (7) Å
*b* = 16.247 (7) Å
*c* = 12.231 (5) Å
β = 111.624 (10)°
*V* = 3664 (3) Å^3^
*Z* = 8
Mo *K*α radiation
μ = 0.10 mm^−1^
*T* = 298 (2) K
0.58 × 0.43 × 0.40 mm

#### Data collection

Bruker SMART CCD area-detector diffractometer
Absorption correction: multi-scan (*SADABS*; Bruker, 1999 ▶) *T*_min_ = 0.945, *T*_max_ = 0.962
9508 measured reflections
3235 independent reflections
1967 reflections with *I* > 2σ(*I*)
*R*_int_ = 0.053

#### Refinement

*R*[*F* ^2^ > 2σ(*F* ^2^)] = 0.064
*wR*(*F* ^2^) = 0.203
*S* = 1.04
3235 reflections
487 parameters
H-atom parameters constrained
Δρ_max_ = 0.42 e Å^−3^
Δρ_min_ = −0.23 e Å^−3^

### 

Data collection: *SMART* (Bruker, 2001 ▶); cell refinement: *SAINT* (Bruker, 2001 ▶); data reduction: *SAINT*; program(s) used to solve structure: *SHELXS97* (Sheldrick, 2008 ▶); program(s) used to refine structure: *SHELXL97* (Sheldrick, 2008 ▶); molecular graphics: *ORTEP-3 for Windows* (Farrugia, 1997 ▶); software used to prepare material for publication: *SHELXTL* (Sheldrick, 2008 ▶).

## Supplementary Material

Crystal structure: contains datablocks I, global. DOI: 10.1107/S1600536808042608/hk2548sup1.cif

Structure factors: contains datablocks I. DOI: 10.1107/S1600536808042608/hk2548Isup2.hkl

Additional supplementary materials:  crystallographic information; 3D view; checkCIF report

## Figures and Tables

**Table 1:** Selected bond lengths (Å)

B1—O1	1.515 (10)
B1—O3	1.429 (9)
B1—O4	1.485 (10)
B1—O6	1.422 (10)
B2—O7	1.477 (10)
B2—O9	1.439 (10)
B2—O10	1.505 (10)
B2—O12	1.421 (10)

**Table 2:** Hydrogen-bond geometry (Å, °)

*D*—H⋯*A*	*D*—H	H⋯*A*	*D*⋯*A*	*D*—H⋯*A*
N1—H1*A*⋯O8	0.90	1.95	2.828 (9)	163
N1—H1*B*⋯O2	0.90	1.93	2.829 (9)	174
N2—H2*A*⋯O5^i^	0.90	1.96	2.824 (10)	159
N2—H2*B*⋯O11	0.90	1.98	2.855 (9)	163

Symmetry code: (i) 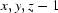.

**Fig. 1.**
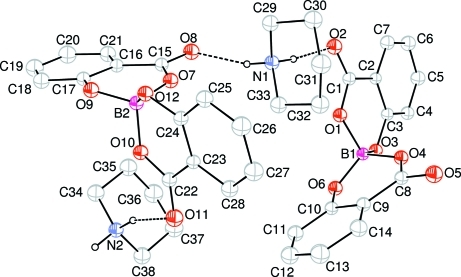
The molecular structure of the title molecule, with the atom-numbering scheme. Displacement ellipsoids are drawn at the 20% probability level. Hydrogen bonds are shown as dashed lines.

**Fig. 2.**
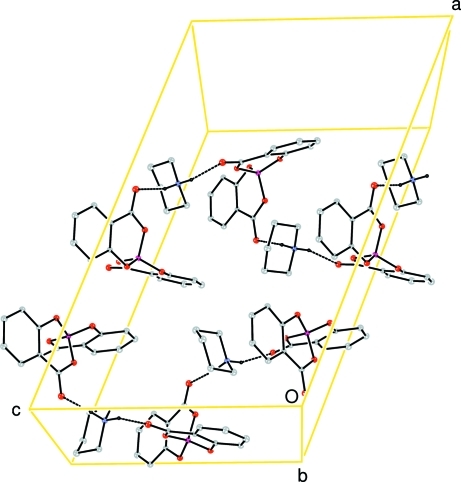
A partial packing diagram of the title compound. Hydrogen bonds are shown as dashed lines.

## supplementary crystallographic information

### Comment

To date, alkali-metals bis(salicylato)borates have received the most attention (Zhang *et al.*, 2005; Downard *et al.*, 2002), since the lithium organoborates had been considered as the lithium battery electrolytes (Barthel *et al.*, 2000). In contrast, studies of organic base bis(salicylato)- borates have been less extensive (Li & Liu, 2006; Han *et al.*, 2007). We report herein the synthesis and crystal structure of the title compound.

The asymmetric unit of the title compound contains two [C_5_H_12_N]^+^ cations and two [BO_4_(C_7_H_4_O)_2_]^-^ anions (Fig.1). In the anions, the *sp*^3^-hybridized B atoms are bonded to four oxygen atoms in distorted tetrahedral geometries (Table 1). Rings B (C2-C7), D (C9-C14) and F (C16-C21), H (C23-C28) are, of course, planar, and they are oriented at dihedral angles of B/D = 76.27 (3)° and D/F = 83.86 (3)°. Rings A (B1/O1/O3/C1-C3), C (B1/O4/O6/C8-C10) and E (B2/O7/O9/C15-C17), G (B2/O10/O12/C22-C24) are not planar, having total puckering amplitudes, Q_T_, of 0.739 (2), 0.689 (3) Å and 0.724 (3), 0.859 (3) Å, respectively, twisted-boat conformations [φ = -52.97 (4)° and θ = 105.16 (5)°; φ = -51.39 (4)° and θ = 104.63 (3)°; φ = -56.94 (4)° and θ = 109.52 (5)°; φ = -55.15 (5)° and θ = 108.00 (5)°, respectively] (Cremer & Pople, 1975). Rings I (N1/C29-C33) and J (N2/C34-C38) adopt, of course, chair conformations, having total puckering amplitudes, Q_T_, of 0.562 (3) and 0.562 (3) Å, respectively [φ = -69.55 (3)° and θ = 178.57 (4)°; φ = -132.85 (4)° and θ = 4.37 (4)°, respectively] (Cremer & Pople, 1975). The intramolecular N-H···O hydrogen bonds (Table 2 and Fig. 1) link the cations to the anions.

In the crystal structure, the intra- and intermolecular N-H···O hydrogen bonds (Table 2) link the molecules (Fig. 2), in which they may be effective in the stabilization of the structure.

### Experimental

For the preparation of the title compound, a solution of boric acid (0.325 g) in distilled water (5 ml) was added to a stirred solution of salicylic acid (1.418 g) in an ethanol/water (1:1) solvent (10 ml). The reaction mixture was stirred at 353 K for 20 min, and then piperidinium (1 ml) was added slowly. After 4 h continued heating and stirring, the pH of the mixture had changed from 2 to 6, and the clear solution was then allowed to stand for 15 d at room temperature. The title compound was isolated as colorless transparent crystals. Elemental analysis calc.: C 61.80, N 3.79, H 5.47%; found: C 62.12, N 3.62, H 5.12%.

### Refinement

H atoms were positioned geometrically, with N-H = 0.90 Å (for NH_2_) and C-H = 0.93 and 0.97 Å for aromatic and methylene H, respectively, and constrained to ride on their parent atoms with U_iso_(H) = 1.2U_eq_(C,N).

### Crystal data

**Table:**

C_5_H_12_N^+^·C_14_H_8_BO_6_^−^	*F*(000) = 1552
*M**_r_* = 369.17	*D*_x_ = 1.338 Mg m^−^^3^
Monoclinic, *C**c*	Mo *K*α radiation, λ = 0.71073 Å
Hall symbol: C -2yc	Cell parameters from 1899 reflections
*a* = 19.835 (7) Å	θ = 2.2–23.1°
*b* = 16.247 (7) Å	µ = 0.10 mm^−^^1^
*c* = 12.231 (5) Å	*T* = 298 K
β = 111.624 (10)°	Clubbed, colorless
*V* = 3664 (3) Å^3^	0.58 × 0.43 × 0.40 mm
*Z* = 8	

### Data collection

**Table:**

Bruker SMART CCD area-detector diffractometer	3235 independent reflections
Radiation source: fine-focus sealed tube	1967 reflections with *I* > 2σ(*I*)
graphite	*R*_int_ = 0.053
φ and ω scans	θ_max_ = 25.0°, θ_min_ = 1.7°
Absorption correction: multi-scan (*SADABS*; Bruker, 1999)	*h* = −23→23
*T*_min_ = 0.945, *T*_max_ = 0.962	*k* = −19→16
9508 measured reflections	*l* = −14→14

### Refinement

**Table:**

Refinement on *F*^2^	Secondary atom site location: difference Fourier map
Least-squares matrix: full	Hydrogen site location: inferred from neighbouring sites
*R*[*F*^2^ > 2σ(*F*^2^)] = 0.064	H-atom parameters constrained
*wR*(*F*^2^) = 0.203	*w* = 1/[σ^2^(*F*_o_^2^) + (0.1245*P*)^2^] where *P* = (*F*_o_^2^ + 2*F*_c_^2^)/3
*S* = 1.04	(Δ/σ)_max_ < 0.001
3235 reflections	Δρ_max_ = 0.42 e Å^−^^3^
487 parameters	Δρ_min_ = −0.23 e Å^−^^3^
Primary atom site location: structure-invariant direct methods	

### Special details

**Table:**

Geometry. All e.s.d.'s (except the e.s.d. in the dihedral angle between two l.s. planes) are estimated using the full covariance matrix. The cell e.s.d.'s are taken into account individually in the estimation of e.s.d.'s in distances, angles and torsion angles; correlations between e.s.d.'s in cell parameters are only used when they are defined by crystal symmetry. An approximate (isotropic) treatment of cell e.s.d.'s is used for estimating e.s.d.'s involving l.s. planes.
Refinement. Refinement of *F*^2^ against ALL reflections. The weighted *R*-factor *wR* and goodness of fit *S* are based on *F*^2^, conventional *R*-factors *R* are based on *F*, with *F* set to zero for negative *F*^2^. The threshold expression of *F*^2^ > σ(*F*^2^) is used only for calculating *R*-factors(gt) *etc*. and is not relevant to the choice of reflections for refinement. *R*-factors based on *F*^2^ are statistically about twice as large as those based on *F*, and *R*- factors based on ALL data will be even larger.

### Fractional atomic coordinates and isotropic or equivalent isotropic displacement parameters (Å^2^)

**Table:**

	*x*	*y*	*z*	*U*_iso_*/*U*_eq_	
O1	0.2375 (3)	0.3316 (3)	0.5857 (5)	0.0459 (13)	
O2	0.1339 (3)	0.3910 (3)	0.5637 (5)	0.0572 (15)	
O3	0.3536 (3)	0.3847 (3)	0.7220 (5)	0.0458 (13)	
O4	0.3144 (3)	0.2518 (3)	0.7485 (5)	0.0461 (13)	
O5	0.2966 (4)	0.1240 (3)	0.7940 (6)	0.0635 (16)	
O6	0.3499 (3)	0.2835 (3)	0.5839 (5)	0.0490 (13)	
O7	0.0436 (3)	0.2515 (3)	0.0932 (4)	0.0481 (13)	
O8	0.0554 (3)	0.3784 (3)	0.1602 (5)	0.0583 (15)	
O9	0.0048 (3)	0.2223 (3)	−0.1159 (4)	0.0498 (13)	
O10	0.1147 (3)	0.1677 (3)	0.0174 (5)	0.0461 (13)	
O11	0.2152 (3)	0.0991 (4)	0.1091 (6)	0.0630 (16)	
O12	−0.0013 (3)	0.1207 (3)	0.0170 (5)	0.0455 (13)	
N1	0.0823 (3)	0.2848 (4)	0.3669 (6)	0.0476 (16)	
H1A	0.0834	0.3148	0.3057	0.057*	
H1B	0.1011	0.3157	0.4322	0.057*	
N2	0.2593 (3)	0.2086 (4)	−0.0346 (6)	0.0494 (16)	
H2A	0.2611	0.1741	−0.0912	0.059*	
H2B	0.2379	0.1816	0.0081	0.059*	
B1	0.3161 (4)	0.3137 (5)	0.6602 (7)	0.0375 (19)	
B2	0.0379 (5)	0.1900 (5)	0.0013 (8)	0.041 (2)	
C1	0.2001 (4)	0.3872 (4)	0.6166 (7)	0.0429 (18)	
C2	0.2406 (4)	0.4427 (4)	0.7122 (6)	0.0365 (16)	
C3	0.3163 (4)	0.4403 (4)	0.7615 (6)	0.0407 (17)	
C4	0.3549 (4)	0.4981 (5)	0.8441 (7)	0.049 (2)	
H4	0.4053	0.4978	0.8731	0.059*	
C5	0.3183 (5)	0.5560 (5)	0.8832 (8)	0.060 (2)	
H5	0.3439	0.5942	0.9400	0.072*	
C6	0.2431 (5)	0.5571 (5)	0.8373 (8)	0.062 (2)	
H6	0.2183	0.5959	0.8642	0.074*	
C7	0.2053 (5)	0.5024 (5)	0.7539 (8)	0.055 (2)	
H7	0.1549	0.5045	0.7238	0.066*	
C8	0.3096 (4)	0.1710 (4)	0.7260 (7)	0.0429 (18)	
C9	0.3228 (4)	0.1448 (5)	0.6222 (7)	0.0452 (18)	
C10	0.3428 (4)	0.2031 (4)	0.5546 (7)	0.0417 (17)	
C11	0.3556 (5)	0.1784 (6)	0.4551 (8)	0.058 (2)	
H11	0.3693	0.2165	0.4103	0.069*	
C12	0.3480 (5)	0.0977 (6)	0.4244 (9)	0.070 (3)	
H12	0.3559	0.0816	0.3572	0.085*	
C13	0.3292 (6)	0.0393 (6)	0.4874 (10)	0.079 (3)	
H13	0.3243	−0.0154	0.4634	0.095*	
C14	0.3173 (5)	0.0624 (5)	0.5885 (9)	0.064 (2)	
H14	0.3056	0.0228	0.6336	0.077*	
C15	0.0430 (4)	0.3318 (4)	0.0751 (7)	0.0418 (17)	
C16	0.0305 (4)	0.3602 (5)	−0.0449 (7)	0.0420 (17)	
C17	0.0130 (4)	0.3028 (5)	−0.1354 (6)	0.0435 (18)	
C18	0.0028 (4)	0.3290 (6)	−0.2479 (7)	0.058 (2)	
H18	−0.0073	0.2911	−0.3088	0.070*	
C19	0.0076 (5)	0.4118 (6)	−0.2696 (9)	0.074 (3)	
H19	0.0002	0.4294	−0.3455	0.089*	
C20	0.0233 (6)	0.4689 (6)	−0.1802 (9)	0.078 (3)	
H20	0.0256	0.5247	−0.1955	0.094*	
C21	0.0355 (5)	0.4415 (5)	−0.0671 (9)	0.061 (2)	
H21	0.0472	0.4792	−0.0056	0.074*	
C22	0.1505 (4)	0.1094 (4)	0.0904 (7)	0.0451 (19)	
C23	0.1087 (4)	0.0581 (4)	0.1416 (6)	0.0384 (17)	
C24	0.0343 (4)	0.0664 (4)	0.1038 (6)	0.0411 (18)	
C25	−0.0054 (5)	0.0182 (5)	0.1517 (8)	0.053 (2)	
H25	−0.0554	0.0246	0.1278	0.064*	
C26	0.0304 (6)	−0.0395 (5)	0.2353 (8)	0.067 (3)	
H26	0.0041	−0.0731	0.2665	0.080*	
C27	0.1045 (5)	−0.0483 (5)	0.2735 (9)	0.067 (3)	
H27	0.1276	−0.0874	0.3306	0.080*	
C28	0.1445 (5)	0.0001 (5)	0.2280 (7)	0.054 (2)	
H28	0.1946	−0.0056	0.2543	0.065*	
C29	0.0057 (5)	0.2642 (5)	0.3479 (8)	0.057 (2)	
H29A	−0.0162	0.2351	0.2739	0.069*	
H29B	−0.0216	0.3144	0.3436	0.069*	
C30	0.0024 (5)	0.2116 (6)	0.4465 (9)	0.070 (3)	
H30A	−0.0475	0.1962	0.4310	0.084*	
H30B	0.0201	0.2426	0.5193	0.084*	
C31	0.0479 (6)	0.1345 (6)	0.4591 (10)	0.078 (3)	
H31A	0.0271	0.1004	0.3896	0.094*	
H31B	0.0481	0.1030	0.5266	0.094*	
C32	0.1256 (5)	0.1579 (6)	0.4749 (9)	0.070 (3)	
H32A	0.1480	0.1866	0.5491	0.084*	
H32B	0.1533	0.1083	0.4776	0.084*	
C33	0.1276 (5)	0.2104 (6)	0.3794 (8)	0.060 (2)	
H33A	0.1103	0.1796	0.3063	0.073*	
H33B	0.1773	0.2267	0.3947	0.073*	
C34	0.2141 (5)	0.2807 (6)	−0.0911 (9)	0.068 (3)	
H34A	0.1647	0.2630	−0.1338	0.082*	
H34B	0.2325	0.3053	−0.1468	0.082*	
C35	0.2150 (5)	0.3438 (6)	0.0000 (10)	0.073 (3)	
H35A	0.1896	0.3219	0.0478	0.087*	
H35B	0.1894	0.3927	−0.0394	0.087*	
C36	0.2913 (7)	0.3672 (7)	0.0786 (12)	0.090 (4)	
H36A	0.3155	0.3944	0.0326	0.109*	
H36B	0.2897	0.4050	0.1389	0.109*	
C37	0.3326 (6)	0.2910 (6)	0.1350 (10)	0.082 (3)	
H37A	0.3098	0.2658	0.1845	0.098*	
H37B	0.3818	0.3059	0.1843	0.098*	
C38	0.3344 (5)	0.2300 (6)	0.0428 (9)	0.068 (3)	
H38A	0.3599	0.2538	−0.0038	0.082*	
H38B	0.3601	0.1807	0.0806	0.082*	

### Atomic displacement parameters (Å^2^)

**Table:**

	*U*^11^	*U*^22^	*U*^33^	*U*^12^	*U*^13^	*U*^23^
O1	0.050 (3)	0.037 (3)	0.044 (3)	0.001 (2)	0.008 (2)	−0.007 (2)
O2	0.044 (3)	0.059 (3)	0.055 (4)	0.005 (2)	0.002 (3)	−0.013 (3)
O3	0.042 (3)	0.033 (3)	0.059 (4)	−0.004 (2)	0.015 (3)	−0.008 (2)
O4	0.060 (3)	0.042 (3)	0.038 (3)	0.006 (2)	0.021 (3)	0.005 (2)
O5	0.092 (4)	0.048 (3)	0.061 (4)	−0.006 (3)	0.042 (3)	0.008 (3)
O6	0.065 (3)	0.036 (3)	0.056 (4)	0.006 (2)	0.033 (3)	0.000 (2)
O7	0.074 (4)	0.034 (3)	0.033 (3)	0.007 (2)	0.016 (3)	0.003 (2)
O8	0.085 (4)	0.042 (3)	0.047 (4)	−0.003 (3)	0.023 (3)	−0.006 (3)
O9	0.066 (3)	0.041 (3)	0.032 (3)	0.002 (2)	0.005 (2)	0.001 (2)
O10	0.052 (3)	0.040 (3)	0.050 (3)	0.005 (2)	0.024 (3)	0.008 (2)
O11	0.054 (4)	0.065 (4)	0.075 (4)	0.006 (3)	0.029 (3)	0.018 (3)
O12	0.050 (3)	0.036 (3)	0.046 (3)	−0.003 (2)	0.012 (3)	0.004 (2)
N1	0.050 (4)	0.058 (4)	0.030 (3)	−0.007 (3)	0.009 (3)	0.000 (3)
N2	0.045 (4)	0.062 (4)	0.041 (4)	−0.004 (3)	0.016 (3)	−0.001 (3)
B1	0.048 (5)	0.032 (4)	0.034 (5)	0.001 (3)	0.017 (4)	−0.004 (3)
B2	0.054 (5)	0.033 (4)	0.033 (5)	0.001 (4)	0.013 (4)	−0.003 (4)
C1	0.046 (5)	0.043 (4)	0.036 (4)	0.000 (3)	0.010 (4)	0.003 (3)
C2	0.042 (4)	0.032 (4)	0.031 (4)	0.000 (3)	0.008 (3)	0.003 (3)
C3	0.046 (4)	0.031 (4)	0.040 (5)	0.002 (3)	0.009 (4)	0.003 (3)
C4	0.049 (5)	0.047 (4)	0.041 (5)	−0.006 (3)	0.004 (4)	−0.012 (4)
C5	0.079 (6)	0.045 (5)	0.044 (5)	−0.002 (4)	0.010 (4)	−0.019 (4)
C6	0.064 (6)	0.061 (5)	0.055 (6)	0.016 (4)	0.015 (5)	−0.016 (4)
C7	0.052 (5)	0.050 (5)	0.054 (5)	0.005 (4)	0.010 (4)	−0.010 (4)
C8	0.045 (4)	0.040 (4)	0.043 (5)	0.000 (3)	0.016 (4)	0.001 (4)
C9	0.050 (4)	0.041 (4)	0.045 (5)	0.005 (3)	0.017 (4)	−0.005 (4)
C10	0.041 (4)	0.042 (4)	0.044 (5)	0.005 (3)	0.018 (3)	−0.003 (4)
C11	0.060 (5)	0.073 (6)	0.052 (6)	−0.002 (4)	0.033 (5)	−0.003 (5)
C12	0.073 (6)	0.081 (7)	0.060 (6)	−0.002 (5)	0.027 (5)	−0.025 (5)
C13	0.091 (8)	0.055 (6)	0.095 (8)	−0.006 (5)	0.037 (7)	−0.035 (6)
C14	0.072 (6)	0.044 (5)	0.074 (7)	−0.006 (4)	0.025 (5)	−0.006 (4)
C15	0.052 (4)	0.036 (4)	0.038 (4)	0.002 (3)	0.018 (4)	−0.003 (3)
C16	0.045 (4)	0.040 (4)	0.040 (4)	0.000 (3)	0.013 (3)	0.003 (3)
C17	0.043 (4)	0.054 (5)	0.029 (4)	0.005 (4)	0.007 (3)	0.007 (4)
C18	0.056 (5)	0.083 (6)	0.026 (4)	−0.002 (4)	0.005 (4)	0.006 (4)
C19	0.091 (7)	0.078 (7)	0.047 (6)	−0.010 (5)	0.019 (5)	0.026 (5)
C20	0.099 (8)	0.061 (6)	0.068 (7)	−0.008 (5)	0.023 (6)	0.030 (6)
C21	0.079 (6)	0.040 (5)	0.063 (6)	−0.008 (4)	0.024 (5)	0.004 (4)
C22	0.047 (5)	0.043 (4)	0.043 (5)	0.002 (3)	0.015 (4)	−0.004 (3)
C23	0.046 (4)	0.034 (4)	0.038 (4)	0.003 (3)	0.018 (3)	−0.001 (3)
C24	0.051 (5)	0.033 (4)	0.036 (4)	−0.001 (3)	0.012 (4)	−0.004 (3)
C25	0.059 (5)	0.052 (5)	0.053 (5)	−0.008 (4)	0.024 (4)	0.000 (4)
C26	0.096 (8)	0.052 (5)	0.059 (6)	−0.007 (5)	0.038 (6)	0.013 (4)
C27	0.075 (6)	0.059 (5)	0.065 (6)	0.019 (5)	0.024 (5)	0.031 (5)
C28	0.064 (6)	0.055 (5)	0.040 (5)	0.013 (4)	0.015 (4)	0.008 (4)
C29	0.055 (5)	0.070 (5)	0.045 (5)	0.011 (4)	0.017 (4)	−0.001 (4)
C30	0.057 (5)	0.095 (7)	0.058 (6)	−0.010 (5)	0.022 (5)	−0.009 (5)
C31	0.092 (8)	0.069 (6)	0.070 (7)	−0.012 (5)	0.025 (6)	0.013 (5)
C32	0.067 (6)	0.061 (6)	0.069 (7)	0.003 (4)	0.011 (5)	−0.001 (5)
C33	0.051 (5)	0.073 (6)	0.049 (5)	0.006 (4)	0.008 (4)	−0.013 (5)
C34	0.060 (5)	0.079 (6)	0.065 (6)	0.003 (5)	0.022 (5)	0.022 (5)
C35	0.071 (6)	0.065 (6)	0.082 (7)	0.000 (5)	0.030 (5)	−0.004 (5)
C36	0.093 (8)	0.072 (7)	0.118 (11)	−0.017 (6)	0.053 (8)	−0.023 (7)
C37	0.066 (6)	0.083 (7)	0.079 (8)	0.000 (5)	0.007 (6)	−0.036 (6)
C38	0.052 (5)	0.075 (6)	0.069 (7)	−0.003 (4)	0.011 (5)	−0.010 (5)

### Geometric parameters (Å, °)

**Table:**

O1—C1	1.310 (9)	C15—C16	1.470 (11)
O1—B1	1.515 (10)	C16—C21	1.359 (11)
O2—C1	1.233 (9)	C16—C17	1.389 (10)
O3—C3	1.364 (8)	C17—C18	1.383 (11)
O3—B1	1.429 (9)	C18—C19	1.381 (13)
O4—C8	1.336 (8)	C18—H18	0.9300
O4—B1	1.485 (10)	C19—C20	1.378 (14)
O5—C8	1.224 (9)	C19—H19	0.9300
O6—C10	1.347 (8)	C20—C21	1.387 (13)
O6—B1	1.422 (10)	C20—H20	0.9300
O7—C15	1.323 (8)	C21—H21	0.9300
O7—B2	1.477 (10)	C22—C23	1.469 (10)
O8—C15	1.236 (9)	C23—C24	1.380 (10)
O9—C17	1.349 (9)	C23—C28	1.399 (10)
O9—B2	1.439 (10)	C24—C25	1.385 (11)
O10—C22	1.316 (9)	C25—C26	1.375 (12)
O10—B2	1.505 (10)	C25—H25	0.9300
O11—C22	1.229 (9)	C26—C27	1.374 (13)
O12—C24	1.360 (9)	C26—H26	0.9300
O12—B2	1.421 (10)	C27—C28	1.372 (12)
N1—C29	1.489 (10)	C27—H27	0.9300
N1—C33	1.480 (10)	C28—H28	0.9300
N1—H1A	0.9000	C29—C30	1.499 (13)
N1—H1B	0.9000	C29—H29A	0.9700
N2—C34	1.482 (11)	C29—H29B	0.9700
N2—C38	1.483 (11)	C30—C31	1.518 (14)
N2—H2A	0.9000	C30—H30A	0.9700
N2—H2B	0.9000	C30—H30B	0.9700
C1—C2	1.461 (10)	C31—C32	1.528 (14)
C2—C3	1.397 (10)	C31—H31A	0.9700
C2—C7	1.398 (10)	C31—H31B	0.9700
C3—C4	1.385 (10)	C32—C33	1.458 (13)
C4—C5	1.377 (12)	C32—H32A	0.9700
C4—H4	0.9300	C32—H32B	0.9700
C5—C6	1.387 (13)	C33—H33A	0.9700
C5—H5	0.9300	C33—H33B	0.9700
C6—C7	1.352 (12)	C34—C35	1.510 (14)
C6—H6	0.9300	C34—H34A	0.9700
C7—H7	0.9300	C34—H34B	0.9700
C8—C9	1.452 (11)	C35—C36	1.512 (16)
C9—C14	1.393 (11)	C35—H35A	0.9700
C9—C10	1.407 (11)	C35—H35B	0.9700
C10—C11	1.392 (11)	C36—C37	1.504 (16)
C11—C12	1.357 (12)	C36—H36A	0.9700
C11—H11	0.9300	C36—H36B	0.9700
C12—C13	1.359 (14)	C37—C38	1.512 (14)
C12—H12	0.9300	C37—H37A	0.9700
C13—C14	1.393 (14)	C37—H37B	0.9700
C13—H13	0.9300	C38—H38A	0.9700
C14—H14	0.9300	C38—H38B	0.9700
			
C33—N1—C29	112.2 (6)	C18—C19—H19	119.5
C33—N1—H1A	109.2	C19—C20—C21	118.7 (8)
C29—N1—H1A	109.2	C19—C20—H20	120.6
C33—N1—H1B	109.2	C21—C20—H20	120.6
C29—N1—H1B	109.2	C16—C21—C20	120.8 (9)
H1A—N1—H1B	107.9	C16—C21—H21	119.6
C34—N2—C38	113.8 (7)	C20—C21—H21	119.6
C34—N2—H2A	108.8	O11—C22—O10	119.4 (7)
C38—N2—H2A	108.8	O11—C22—C23	124.0 (7)
C34—N2—H2B	108.8	O10—C22—C23	116.6 (6)
C38—N2—H2B	108.8	C24—C23—C28	120.1 (7)
H2A—N2—H2B	107.7	C24—C23—C22	120.3 (6)
C1—O1—B1	121.7 (6)	C28—C23—C22	119.7 (7)
C3—O3—B1	118.5 (6)	O12—C24—C23	120.6 (6)
C8—O4—B1	122.3 (6)	O12—C24—C25	118.9 (7)
C10—O6—B1	118.7 (6)	C23—C24—C25	120.5 (7)
C15—O7—B2	123.1 (6)	C26—C25—C24	118.7 (8)
C17—O9—B2	119.3 (6)	C26—C25—H25	120.6
C22—O10—B2	122.0 (6)	C24—C25—H25	120.6
C24—O12—B2	117.8 (6)	C27—C26—C25	121.3 (8)
O6—B1—O3	110.3 (6)	C27—C26—H26	119.4
O6—B1—O4	112.7 (6)	C25—C26—H26	119.4
O3—B1—O4	108.0 (6)	C28—C27—C26	120.4 (8)
O6—B1—O1	107.5 (6)	C28—C27—H27	119.8
O3—B1—O1	112.5 (6)	C26—C27—H27	119.8
O4—B1—O1	105.9 (6)	C27—C28—C23	119.0 (8)
O12—B2—O9	110.7 (6)	C27—C28—H28	120.5
O12—B2—O7	108.9 (7)	C23—C28—H28	120.5
O9—B2—O7	113.0 (6)	N1—C29—C30	110.3 (7)
O12—B2—O10	111.7 (6)	N1—C29—H29A	109.6
O9—B2—O10	106.7 (6)	C30—C29—H29A	109.6
O7—B2—O10	105.9 (6)	N1—C29—H29B	109.6
O2—C1—O1	119.4 (7)	C30—C29—H29B	109.6
O2—C1—C2	123.6 (7)	H29A—C29—H29B	108.1
O1—C1—C2	116.9 (6)	C29—C30—C31	110.6 (8)
C3—C2—C7	117.7 (7)	C29—C30—H30A	109.5
C3—C2—C1	121.0 (6)	C31—C30—H30A	109.5
C7—C2—C1	121.2 (7)	C29—C30—H30B	109.5
O3—C3—C4	118.8 (7)	C31—C30—H30B	109.5
O3—C3—C2	120.2 (6)	H30A—C30—H30B	108.1
C4—C3—C2	120.8 (7)	C30—C31—C32	110.0 (8)
C5—C4—C3	119.8 (8)	C30—C31—H31A	109.7
C5—C4—H4	120.1	C32—C31—H31A	109.7
C3—C4—H4	120.1	C30—C31—H31B	109.7
C4—C5—C6	119.6 (7)	C32—C31—H31B	109.7
C4—C5—H5	120.2	H31A—C31—H31B	108.2
C6—C5—H5	120.2	C33—C32—C31	111.6 (8)
C7—C6—C5	120.7 (8)	C33—C32—H32A	109.3
C7—C6—H6	119.6	C31—C32—H32A	109.3
C5—C6—H6	119.6	C33—C32—H32B	109.3
C6—C7—C2	121.2 (8)	C31—C32—H32B	109.3
C6—C7—H7	119.4	H32A—C32—H32B	108.0
C2—C7—H7	119.4	C32—C33—N1	111.4 (7)
O5—C8—O4	119.3 (7)	C32—C33—H33A	109.3
O5—C8—C9	124.2 (7)	N1—C33—H33A	109.3
O4—C8—C9	116.5 (7)	C32—C33—H33B	109.3
C14—C9—C10	118.9 (7)	N1—C33—H33B	109.3
C14—C9—C8	121.4 (8)	H33A—C33—H33B	108.0
C10—C9—C8	119.8 (6)	N2—C34—C35	110.7 (8)
O6—C10—C11	118.5 (7)	N2—C34—H34A	109.5
O6—C10—C9	121.4 (7)	C35—C34—H34A	109.5
C11—C10—C9	120.1 (7)	N2—C34—H34B	109.5
C12—C11—C10	118.9 (9)	C35—C34—H34B	109.5
C12—C11—H11	120.6	H34A—C34—H34B	108.1
C10—C11—H11	120.6	C34—C35—C36	112.1 (8)
C11—C12—C13	123.0 (9)	C34—C35—H35A	109.2
C11—C12—H12	118.5	C36—C35—H35A	109.2
C13—C12—H12	118.5	C34—C35—H35B	109.2
C12—C13—C14	119.1 (8)	C36—C35—H35B	109.2
C12—C13—H13	120.4	H35A—C35—H35B	107.9
C14—C13—H13	120.4	C37—C36—C35	109.5 (8)
C9—C14—C13	120.0 (9)	C37—C36—H36A	109.8
C9—C14—H14	120.0	C35—C36—H36A	109.8
C13—C14—H14	120.0	C37—C36—H36B	109.8
O8—C15—O7	118.4 (7)	C35—C36—H36B	109.8
O8—C15—C16	123.8 (7)	H36A—C36—H36B	108.2
O7—C15—C16	117.7 (6)	C36—C37—C38	110.9 (9)
C21—C16—C17	120.6 (7)	C36—C37—H37A	109.5
C21—C16—C15	120.4 (7)	C38—C37—H37A	109.5
C17—C16—C15	119.0 (6)	C36—C37—H37B	109.5
O9—C17—C18	119.5 (7)	C38—C37—H37B	109.5
O9—C17—C16	121.4 (6)	H37A—C37—H37B	108.1
C18—C17—C16	119.1 (7)	N2—C38—C37	109.6 (7)
C19—C18—C17	119.8 (9)	N2—C38—H38A	109.7
C19—C18—H18	120.1	C37—C38—H38A	109.7
C17—C18—H18	120.1	N2—C38—H38B	109.7
C20—C19—C18	120.9 (8)	C37—C38—H38B	109.7
C20—C19—H19	119.5	H38A—C38—H38B	108.2
			
C10—O6—B1—O3	154.0 (6)	C9—C10—C11—C12	0.6 (12)
C10—O6—B1—O4	33.3 (9)	C10—C11—C12—C13	−0.9 (14)
C10—O6—B1—O1	−83.0 (8)	C11—C12—C13—C14	−0.2 (16)
C3—O3—B1—O6	154.5 (6)	C10—C9—C14—C13	−2.0 (13)
C3—O3—B1—O4	−82.0 (7)	C8—C9—C14—C13	179.0 (9)
C3—O3—B1—O1	34.5 (9)	C12—C13—C14—C9	1.7 (15)
C8—O4—B1—O6	−31.5 (9)	B2—O7—C15—O8	−172.8 (7)
C8—O4—B1—O3	−153.5 (6)	B2—O7—C15—C16	4.7 (10)
C8—O4—B1—O1	85.8 (7)	O8—C15—C16—C21	3.1 (11)
C1—O1—B1—O6	−153.1 (6)	O7—C15—C16—C21	−174.3 (7)
C1—O1—B1—O3	−31.6 (9)	O8—C15—C16—C17	−176.4 (7)
C1—O1—B1—O4	86.2 (7)	O7—C15—C16—C17	6.3 (10)
C24—O12—B2—O9	157.6 (6)	B2—O9—C17—C18	159.2 (7)
C24—O12—B2—O7	−77.7 (7)	B2—O9—C17—C16	−21.7 (11)
C24—O12—B2—O10	38.9 (9)	C21—C16—C17—O9	−177.1 (7)
C17—O9—B2—O12	152.9 (6)	C15—C16—C17—O9	2.3 (11)
C17—O9—B2—O7	30.4 (10)	C21—C16—C17—C18	2.0 (11)
C17—O9—B2—O10	−85.5 (8)	C15—C16—C17—C18	−178.6 (7)
C15—O7—B2—O12	−145.8 (6)	O9—C17—C18—C19	176.8 (8)
C15—O7—B2—O9	−22.4 (10)	C16—C17—C18—C19	−2.3 (12)
C15—O7—B2—O10	94.0 (7)	C17—C18—C19—C20	0.7 (14)
C22—O10—B2—O12	−31.6 (9)	C18—C19—C20—C21	1.2 (16)
C22—O10—B2—O9	−152.6 (6)	C17—C16—C21—C20	−0.1 (13)
C22—O10—B2—O7	86.8 (8)	C15—C16—C21—C20	−179.5 (9)
B1—O1—C1—O2	−168.9 (7)	C19—C20—C21—C16	−1.5 (15)
B1—O1—C1—C2	12.2 (9)	B2—O10—C22—O11	−172.8 (7)
O2—C1—C2—C3	−174.1 (7)	B2—O10—C22—C23	9.4 (9)
O1—C1—C2—C3	4.8 (10)	O11—C22—C23—C24	−171.1 (7)
O2—C1—C2—C7	3.2 (11)	O10—C22—C23—C24	6.6 (10)
O1—C1—C2—C7	−178.0 (7)	O11—C22—C23—C28	8.2 (11)
B1—O3—C3—C4	164.9 (7)	O10—C22—C23—C28	−174.1 (7)
B1—O3—C3—C2	−20.2 (10)	B2—O12—C24—C23	−25.9 (9)
C7—C2—C3—O3	−178.4 (7)	B2—O12—C24—C25	155.6 (7)
C1—C2—C3—O3	−1.0 (10)	C28—C23—C24—O12	−177.8 (6)
C7—C2—C3—C4	−3.6 (10)	C22—C23—C24—O12	1.5 (10)
C1—C2—C3—C4	173.8 (7)	C28—C23—C24—C25	0.7 (11)
O3—C3—C4—C5	178.5 (7)	C22—C23—C24—C25	179.9 (7)
C2—C3—C4—C5	3.6 (12)	O12—C24—C25—C26	176.8 (7)
C3—C4—C5—C6	−1.5 (13)	C23—C24—C25—C26	−1.7 (11)
C4—C5—C6—C7	−0.6 (14)	C24—C25—C26—C27	1.6 (13)
C5—C6—C7—C2	0.6 (14)	C25—C26—C27—C28	−0.6 (14)
C3—C2—C7—C6	1.5 (12)	C26—C27—C28—C23	−0.5 (14)
C1—C2—C7—C6	−175.9 (8)	C24—C23—C28—C27	0.4 (12)
B1—O4—C8—O5	−168.2 (7)	C22—C23—C28—C27	−178.9 (8)
B1—O4—C8—C9	13.7 (10)	C33—N1—C29—C30	56.8 (9)
O5—C8—C9—C14	3.8 (12)	N1—C29—C30—C31	−56.3 (10)
O4—C8—C9—C14	−178.2 (7)	C29—C30—C31—C32	55.3 (11)
O5—C8—C9—C10	−175.1 (8)	C30—C31—C32—C33	−55.2 (12)
O4—C8—C9—C10	2.9 (10)	C31—C32—C33—N1	55.6 (10)
B1—O6—C10—C11	160.6 (7)	C29—N1—C33—C32	−56.7 (9)
B1—O6—C10—C9	−19.4 (10)	C38—N2—C34—C35	−53.5 (10)
C14—C9—C10—O6	−179.1 (7)	N2—C34—C35—C36	52.9 (11)
C8—C9—C10—O6	−0.1 (11)	C34—C35—C36—C37	−55.8 (13)
C14—C9—C10—C11	0.9 (12)	C35—C36—C37—C38	58.2 (12)
C8—C9—C10—C11	179.9 (7)	C34—N2—C38—C37	55.9 (11)
O6—C10—C11—C12	−179.4 (7)	C36—C37—C38—N2	−57.8 (12)

### Hydrogen-bond geometry (Å, °)

**Table:**

*D*—H···*A*	*D*—H	H···*A*	*D*···*A*	*D*—H···*A*
N1—H1A···O8	0.90	1.95	2.828 (9)	163
N1—H1B···O2	0.90	1.93	2.829 (9)	174
N2—H2A···O5^i^	0.90	1.96	2.824 (10)	159
N2—H2B···O11	0.90	1.98	2.855 (9)	163

Symmetry codes: (i) *x*, *y*, *z*−1.
